# 2960. A Gut Pathobiont Triggers Neuroinflammation and Neurocognitive Impairment via Breaching the Gut-Brain Axis in a Preclinical Model of Alzheimer’s Disease: From Microbial Pathogenesis to Neuropathogenesis

**DOI:** 10.1093/ofid/ofad500.199

**Published:** 2023-11-27

**Authors:** Gwoncheol Park, Saurabh Kadyan, Ravinder Nagpal

**Affiliations:** Florida State University, Tallahassee, FL; Florida State University, Tallahassee, FL; Florida State University, Tallahassee, FL

## Abstract

**Background:**

*Klebsiella pneumoniae* (*Kpn*) is notorious for causing nosocomial infections and is commonly found in elderly patients in hospitals with Alzheimer's disease (AD). This bacterium can cause bloodstream infection, which may worsen AD pathophysiology, but there are no studies demonstrating the mechanistic role of this pathogen in AD.

**Methods:**

We infected APP/PS mice intestinally with *Kpn* with (Kpn+Ab) and without antibiotics (Kpn) for a week and then fecal and serum samples were collected 1 and 3 weeks afterwards. Neurocognitive function and motor coordination were tested followed by collection of intestinal and brain tissues at 5 weeks. Microbiome profiles were measured using 16S rRNA amplicon sequencing.

Study design

**Figure:**
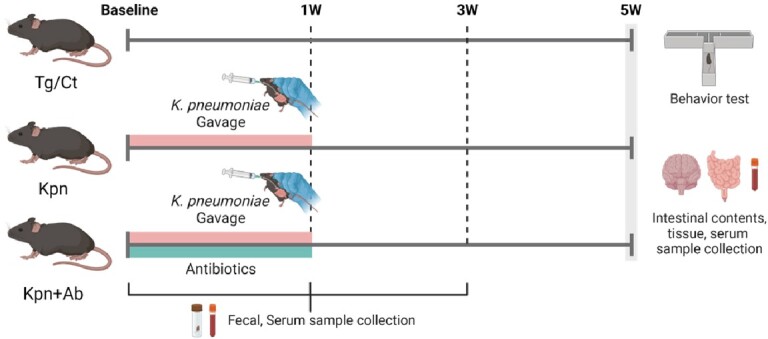

**Results:**

*Kpn* was found to have explosively increased in the gut of all mice that received antibiotics and negatively correlated with microbial diversity. Conversely, *Kpn* was barely detected in the mice that did not receive antibiotics. This underscores the role of antibiotic-induced gut dysbiosis in instigating a ‘pathobiome’ signature. Notably, after five weeks post-infection, *Kpn* was found to be more abundantly colonized in the small intestine than large colon. Importantly, in the Kpn+Ab group, *Kpn* was detected not only in the blood but also in the brain, indicating the translocation of *Kpn* from the intestine to the brain via circulation. In mice in which *Kpn* was found in the brain, a substantial amount of neuroinflammation caused by bacterial infection was observed. Subsequent neurocognitive and behavioral tests revealed that mice infected with *Kpn*, especially those in the Kpn+Ab group, had impaired memory function and motor coordination.

Effects of Klebsiella pneumoniae colonization.

**Figure:**
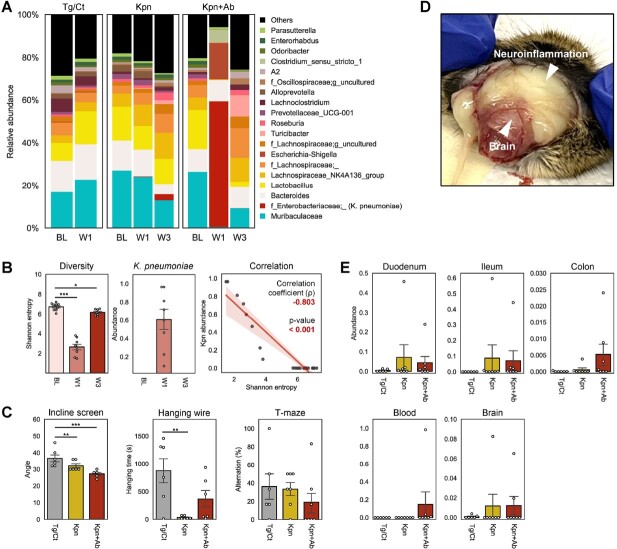

A. Changes in microbial composition before and after K. pn colonization, B. Correlation between microbial diversity (Shannon entropy) and abundance of Kpn of Kpn+Ab group C. Assessment of motor coordination (incline screen and hanging wire test) and memory function (T-maze) test. D. Neuroinflammation after K.pn translocation from gut to brain, E. Abundance of K.pn in the three parts of small intestine, colon, blood, and brain.

**Conclusion:**

These results corroborate the emerging notion of the implicating role of the gut dysbiosis and consequent gut-brain axis impairment in Alzheimer’s neuropathology. Our findings suggest that Alzheimer's patients who are hospitalized and treated with antibiotics are at a higher risk of contracting multi-drug resistant *Kpn*. Further, these data also hint that gut pathobiome may increase the host’s predisposition to AD by breaching the gut-brain axis thereby triggering neuroinflammation and impairing neurocognitive function. Our ongoing studies are anticipated to reveal underlying mechanisms and will be presented at the conference.

**Disclosures:**

**All Authors**: No reported disclosures

